# Findings from RCT’s on virtual reality-based interventions for auditory hallucinations and paranoia

**DOI:** 10.1192/j.eurpsy.2025.55

**Published:** 2025-08-26

**Authors:** L. Birkedal Glenthøj, M. Nordentoft

**Affiliations:** 1 VIRTU Research Group, Mental Health Center Copenhagen, Copenhagen University Hospital; 2 Psychology; 3 Clinical Medicine, University of Copenhagen; 4 Copenhagen Research Center on Mental Health, Copenhagen, Denmark

## Abstract

**Abstract: Introduction:**

Virtual reality (VR) has the potential to enhance current psychotherapies for psychotic symptoms by simulating virtual environments that evoke responses reflective of real-world scenarios.

**Objective:**

This study aimed to evaluate the effectiveness of VR-based psychotherapeutic interventions through findings from two large-scale randomized clinical trials—CHALLENGE and Face Your Fears—that targeted auditory hallucinations (AH) and paranoia, respectively, in individuals with schizophrenia spectrum disorders (SSD).

**Method:**

The CHALLENGE and Face Your Fears trials were randomized, assessor-blinded, parallel-group superiority trials that enrolled 270 and 254 patients with SSD, respectively. In the CHALLENGE trial, participants were randomized to 7 sessions of Challenge-VR therapy (Challenge-VRT) or treatment-as-usual (TAU). In Face Your Fears, participants received either 10 sessions of VR-CBT or standard CBT.

**Results:**

Linear mixed-model analyses on primary and secondary outcomes in both trials revealed that in the CHALLENGE Trial, Challenge-VRT significantly reduced AH symptom severity, as measured by the Psychotic Symptoms Rating Scales (PSYRATS-AH total, adjusted mean difference: -2.26, 95% CI: -4.26 to -0.25, p = 0.03) and frequency (PSYRATS-Frequency, adjusted mean difference: -0.84, 95% CI: -1.53 to -0.14, p = 0.02) at treatment cessation. Face Your Fears Trial: No significant differences were observed between groups on the primary outcome at endpoint (adjusted mean difference: 1.12, 95% CI: -1.75 to 3.99; Cohen’s d = 0.10; p = 0.44). However, both groups demonstrated large within-group improvements (VR-CBT: Cohen’s d = 0.88; standard CBT: Cohen’s d = 0.87). Standard CBT demonstrated superiority over VR-CBT on the secondary outcome measure emotion recognition latency overall at treatment cessation (adjusted mean difference -348.3, 95%CI: -696-6 to -0.04; Cohen’s d = 0.25, p= 0.05), and on emotion recognition accuracy, sadness at 9 months follow-up (adjusted mean difference 0.85, 95% CI: 0.06 to 1.63; Cohen’s d = 0.27, p= 0.03). No serious adverse events were reported in either trial.

**Conclusion:**

Challenge-VRT appears to be a promising treatment for reducing the severity of AH in SSD, though further research is required to optimize and extend its efficacy across broader aspects of schizophrenia. In contrast, VR-CBT did not demonstrate superiority over standard CBT for paranoia, suggesting that both treatments may offer comparable benefits. Future research should explore mediators and moderators of treatment efficacy, as well as patient preferences, to tailor interventions for maximum impact.

**Disclosure of Interest:**

L. Birkedal Glenthøj Consultant of: Consultancy for Khora-VR., M. Nordentoft: None Declared

